# Patient safety and public health concerns: poor dissolution rate of pioglitazone tablets obtained from China, Myanmar and internet sites

**DOI:** 10.1186/s40360-021-00478-x

**Published:** 2021-03-02

**Authors:** Mohammad Sofiqur Rahman, Naoko Yoshida, Hirohito Tsuboi, Erina Maeda, Andrea Vanessa Velasco Ibarra, Theingi Zin, Yoshio Akimoto, Tsuyoshi Tanimoto, Kazuko Kimura

**Affiliations:** 1 Medi-Quality Security Institute, Graduate School of Medical Sciences, Kanazawa University, 920-1192 Kanazawa, Japan; 2 Department of Clinical Pharmacy and Healthcare Sciences, Kanazawa University, 920-1192 Kanazawa, Japan; 3 Department of Food and Drug Administration (FDA), Ministry of Health and Sports, Naypyidaw, Myanmar; 4 Pharmaceutical and Medical Device Regulatory Science Society of Japan, 150-0002 Osaka, Japan

**Keywords:** Medicine, Pharmacoepidemiology, Quality, Substandard, Antidiabetic, Pioglitazone

## Abstract

**Background:**

Poor quality medicines have serious implications for public health. The aim of this study was to explore the quality of the antidiabetic pioglitazone, using samples collected in China and Myanmar, and samples purchased online.

**Methods:**

In this cross-sectional study, we examined samples (*n* = 163) collected from hospitals in Shanghai, China in 2012 (*n* = 44), products purchased via the internet and imported into Japan in 2013 (*n* = 59), and samples purchased in shops in Yangon, Myanmar in 2015 (*n* = 60). Collected samples were subjected to visual inspection, authenticity investigation and quality testing (potency, content uniformity and dissolution test) by high-performance liquid chromatography. Samples were rated as compliant or non-compliant based on the relevant pharmacopoeial acceptance criteria.

**Results:**

Visual inspection of all samples revealed compliant products. However, responses from manufacturers during authenticity investigation were poor. Among the *n* = 44 samples from China, one was non-compliant in the potency test. Among the *n* = 59 samples personally imported into Japan, 38% of generic samples were found to be non-compliant. In Myanmar, 13.3% of samples were non-compliant. Non-compliant samples predominantly failed in the dissolution test. All non-compliant samples were generic.

**Conclusions:**

Despite the apparent satisfactory outcome on the samples from China, pioglitazone samples collected in Myanmar and purchased online for personal import into Japan included many substandard products, which failed quality assessment predominantly because of poor dissolution. Internet providers did not comply with Japanese regulations in various respects.

**Supplementary Information:**

The online version contains supplementary material available at 10.1186/s40360-021-00478-x.

## Background

Substandard and Falsified (SF) medicines are a well-established threat to public health [1–4]. World Health Organization (WHO) defines ‘substandard’ medicines which are often termed as ‘out of specification’ medicines as authorized medical products that fail to meet either their quality standards or specifications, or both. On the other hand, falsified medicines are those that deliberately/fraudulently misrepresent their identity, composition or source [5]. In general, the problem of substandard medicines, as defined by Newton et al. [6, 7], has been overshadowed by the focus on falsified medicines [8–12]. Indeed, the proportion of substandard medicines in circulation is difficult to ascertain because of inadequate reporting. A recent study by the WHO found a failure rate of 10.5% of tested samples [13], while a review by Caudron et al. stated that the percentage of substandard medicines in several Asian and African countries is in the range of 8–46% [9]. A similar study in six African countries revealed that 35% of the collected samples were substandard [12]. Moreover, the problem of substandard medicines is being exacerbated by the rise of online pharmacies [14], which have made drug sub-standardization profitable to unethical manufacturers [15, 16]. Approximately 60% of internet users in Japan and the USA use the internet for health-related activities [17]. Thus, there is a clear risk that international trade in pharmaceuticals via sales on the internet will facilitate the entry of poor-quality products into the legitimate supply chain and for the final users.

In this study, we focused on pioglitazone, which is widely used for the treatment of adult type-2 diabetes mellitus as an adjunct to exercise and diet to improve glycemic control [18]. It is sold in the market as a single product under the brand name Actos or in combination with metformin (Actoplus Met, Actoplus Met XR) and glimepiride (Duetact) [19, 20]. A few reports on the quality of pioglitazone and other medicines from China or Myanmar have appeared [21–23], and provide useful data for comparison with the findings of this study. Additionally, recent reports on the nitrosamine impurities detected in ranitidine and pioglitazone products suggest the presence of scarce quality pioglitazone products in the market [24, 25].

The aim of this work was to assess the quality of pioglitazone circulating in China and Myanmar, as well as that of pioglitazone sold online for personal import. The information obtained here will be of value to public health officials and pharmaceutical practitioners to determine the extent of the problem, and also to provide a baseline for future studies to evaluate interventions designed to improve the drug supply quality, especially in relation to online imports. It will also help guide further research to better understand the health impact of poor-quality medications in these countries.

## Methods

### Ethics approval

Institutional ethical approval was not needed for this study as it is does not involve human subjects, although good ethical practice for such studies has been suggested by Tabernero et al., to maintain the privacy and confidentiality of the surveyors and the surveyed [26]. Regulatory approval was given by the respective countries’ Medicine Regulatory Authorities (MRAs), and annual reports have been submitted to them.

### Study design and sample collection

As suggested by the regulatory authorities of the respective countries, pioglitazone was chosen as a target medicine because of the past history of similar medicines to show problems in the dissolution test. The medicine also appears in the essential drug list of Myanmar. The design and analytical methods used in this study followed as far as possible the guidelines of the WHO and those proposed by Newton et al. [27, 28]. Cross-sectional sampling with the mystery shopper approach was used. In each sampling location, the initial sampling plan was to follow a random sampling protocol, though in practice this was not always possible due to the unavailability of medicine, availability of insufficient quantities, incomplete list of shops, or closure of a listed shop at sampling sites [28, 29]. Samples were collected with prescriptions from hospitals and clinics of Huangpu District and Pudong New Area of Shanghai, China, between October and December in 2012. Personally imported samples purchased via internet sites were collected based on the availability of commercial brands in the site during September and December, 2013. Google Japan was used as a search engine to find sites; the search term was ‘ピオグリタゾン AND 個人輸入’ for Japanese language sites and ‘Pioglitazone and personal import’ for English language sites. In Myanmar, samples were collected without prescription from shops in Yangon during October 2015. The samples were purchased by the mystery shoppers without any preference to specific brands in an attempt to purchase as wide a variety of available commercial brands as possible. Medicines collected from the same shop/site and labeled with the same international non-proprietary name (INN), strength, size, brand name, batch/lot number, and manufacturing and expiry dates were considered as one sample. The maximum number of samples collected from each shop was three.

### Sample analysis

Chemical assessment of the quality of pioglitazone tablets purchased in all the sampling location was carried out at the laboratory of Kanazawa University, Japan. Every sample was placed in an individual ziplock bag together with the recoded data, and securely stored in an air-conditioned laboratory (20–25 °C) until analysis. Analysis of all samples was carried out before the stated expiry date. The analysis consisted of observation, authenticity and legality investigation, registration verification, pharmacopoeial analysis (identification, potency, content uniformity and dissolution test) and dissolution profiling.

### Visual inspection

Each sample was given a unique code after the shipment was received. Details of the packaging condition and label information were noted carefully. Observations included the packaging and labeling, and physical appearance of the tablet (size, shape, color, etc.) according to the WHO guideline and the International Pharmaceutical Federation (FIP) checklist for visual inspection of medicines [28–30]. For personal import samples shipped to Japan from internet pharmacies, the websites were checked for compliance with the Pharmaceutical Affairs Law of Japan [31, 32].

### Authenticity investigation and legitimacy verification

For the authenticity investigation of the products and legitimacy verification of the manufacturers, a detailed questionnaire was sent to each manufacturer and regulatory authority of the manufacturing country. Sample questionnaire for product authentication and legitimacy verification are presented in Supplemental File S1 and Supplemental File S2. Each questionnaire contained detailed information about the product, including manufacturer, batch number, manufacturing and expiry dates, and dosage and strength of the product, as indicated by WHO and other related studies [17, 28, 29, 33]. The registration status of all products as stated on the product packaging was recorded, and included on a questionnaire sent to the importing country to confirm the registration of the product and manufacturer (sample registration verification form is presented as Supplemental File S3).

### Laboratory analysis

Pioglitazone hydrochloride as a reference standard, benzophenone as an internal standard, methanol, acetonitrile, ammonium acetate, potassium chloride and other chemicals of reagent grade were procured from the Wako Pure Chemical Industries Ltd. Japan. Hydrochloric acid was purchased from Nacalai Tesque Inc. and acetic acid from Alfa Aesar. Analysis of the sample was done by high-performance liquid chromatography (HPLC) according to the modified and validated JP (Japanese Pharmacopoeia) protocol [34, 35], using a Shimadzu Prominence HPLC equipped with a Phenomenex Gemini NX C18 column (150 × 4.6 mm) and a UV-photodiode array detector (SPD-20A/20AV Series). The flow rate, injection volume, and detection wavelength were kept unchanged throughout the entire analysis. The dissolution test was performed using 900 mL of a solution for each of the units with an NTR-VS 6P dissolution apparatus (Toyama Sangyo Co. Ltd., Osaka, Japan). The dissolution medium was prepared by mixing 50 mL of 0.2 mol/L hydrochloric acid and 150 mL of potassium chloride solution, adding water to make 1000 mL, and adjusting to pH 2.0 with 5 mol/L hydrochloric acid. Drug release studies were carried out according to the United States Pharmacopoeia (USP) Type II dissolution apparatus paddle method. The paddle was set to rotate at 50 rpm (revolutions per minute) for 45 min and the temperature was maintained at 37 ± 0.50 °C. Standard solutions were prepared by dissolving accurately weighed quantities of pioglitazone hydrochloride (reference standard) and benzophenone (internal standard) in the diluent to obtain concentrations of 0.2 mg/mL and 0.1 mg/mL, respectively. Serial dilutions were made to 0.025 mg/ml. The concentration of the test solution was kept at 0.1 mg/ml. The relationship between the peak area and concentration of each reference standard was linear within the range of 25–200% of the active ingredient (*r*^2^ = 0.999–1.000), and the quality test was performed within that range.

### Compliance criteria

Samples were evaluated as meeting the quality specifications if the amount of active pharmaceutical ingredient (API-pioglitazone hydrochloride) in each of the units, as determined by the content uniformity test, lay within the range of 95.0–105.0% of the label claim. For content uniformity, the acceptance value (AV≦15.0) was calculated according to USP 34 [36]. In the dissolution test, Q = 80% or more was used as the criterion of acceptability as indicated by the pharmacopoeia [34–36].

### Statistical analysis

Descriptive statistical analysis was performed using Microsoft Excel.

## Results

We collected *n* = 44 samples from Shanghai, China, *n* = 60 sample from Yangon, Myanmar and *n* = 59 samples personally imported into Japan. Details of the collected samples are presented in the Table 1.

**Table 1:** Overview of collected samples by sampling site, category and strength

Sampling Site	Year	Category	Strength			
			15 mg	30 mg	45 mg	Total, n
China (Shanghai)	2012	Originator Brand	9	–	–	44
		Generic	35	–	–	
Personal import samples	2013	Originator Brand	19	4	5	59
		Generic	19	9	3	
Yangon, Myanmar	2015	Originator Brand	1	–	–	60
		Generic	59	–	–	
**Total number of samples,** ***n*** **=**			**163**			

### Observational analysis

No unusual or suspicious features were found for any sample during visual inspection of the samples, except for two samples from one manufacturer, where two different batch numbered strips were found in one box. The physical appearance of the samples was also compliant. However, some serious issues were observed with online sites during sample collection. Among the *n* = 32 online sites visited, all were in breach of Japanese regulations in some respect [37]. Site observation results of online pharmacies are summarized in Table 2.

**Table 2:** Observations of internet sites

Category	Number of sites n (%); total, ***n*** = 32
Site without any physical address	6 (18.8%)
Site without contact number	13 (40.0%)
Site without purchasing amount restriction	14 (43.8%)
Site without prescription requirement	32 (100.0%)
Site selling 45 mg pioglitazone (not approved in Japan)	4 (12.5%)
Site delivering a different amount of tablets from that ordered	4 (12.5%)

### Authenticity, legitimacy investigation, and registration verification

The response rate to our questionnaire (Supplemental File S1 and Supplemental File S2) was very low, but the manufacturers who replied confirmed their products to be genuine (Table 3). In the case of manufacturing countries, the best response was found for the personal import samples: 71.4% (5 out of 7) National regulatory authorities (NRAs) confirmed that the manufacturers had approval to manufacture pioglitazone. All the samples collected from Shanghai were found to be registered (Supplemental File S3). Among the *n* = 60 collected samples from Myanmar, one sample was found to be unregistered at Food and Drug Administration (FDA), Myanmar.

**Table 3:** Authenticity investigation and legitimacy verification results of the collected samples

Category	Replies/total number	Reply on samples/number of samples	Authentic, %		
			Yes	No	Unknown^**a**^
**China**					
Manufacturer	1/9	9/35	25.7%	–	74.3%
NRA of the manufacturing country	1/2	9/35	25.7%	–	74.3%
**Personal import samples**					
Manufacturer	1/11	28/59	47.5%	–	52.5%
NRA of the manufacturing country	5/7	37/59	62.7%	–	37.3%
**Myanmar**					
Manufacturer	2/6	9/60	15.0%%	–	85.0%
NRA of the manufacturing country	1/4	1/60	1.7%	–	98.3%

^a^It was not possible to check if the samples were genuine or the manufacturers were legitimate

### Results of laboratory analysis

**Fig. 1:**
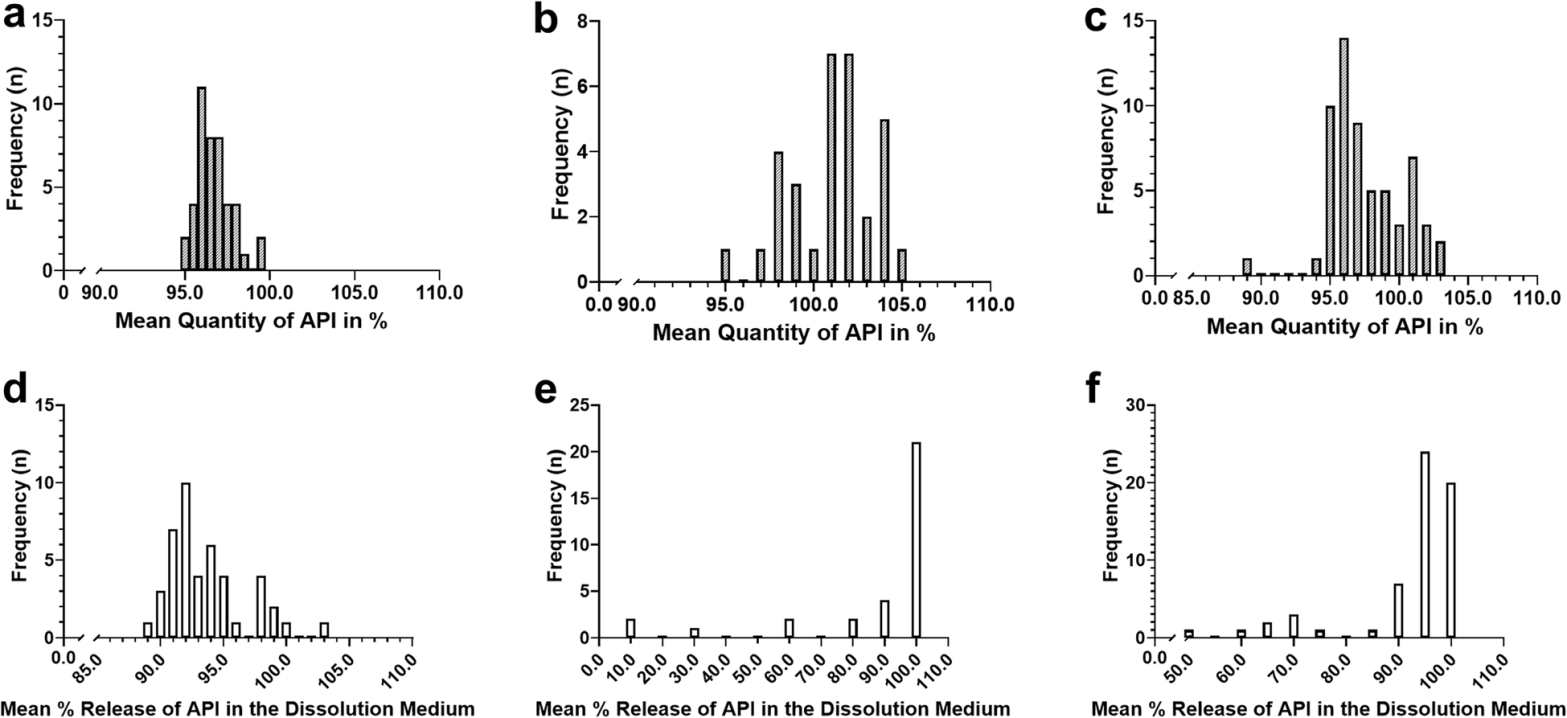
Frequency of the Mean %API of Samples in the Potency Test and Mean %API of Samples Dissolved in the Dissolution Medium in the Dissolution Test. **a** Frequency of %API of samples from Shanghai, China in 2012; **b** Frequency of %API of samples from personal import to Japan in 2013; **c** Frequency of %API of samples from Yangon, Myanmar in 2015; **d** Frequency of mean %API of samples from Shanghai, China dissolved in the dissolution medium; **e** Frequency of mean %API of samples from personal import dissolved in the dissolution medium; and **f** Frequency of mean %API of samples from Yangon, Myanmar dissolved in the dissolution medium

The results of the identification test are not shown in the table, as all the samples were confirmed to contain pioglitazone. Quantitative analysis by HPLC showed that all samples were within the compliance range (95–105%), except for one sample from China (1 out of *n* = 44 samples) among the total of *n* = 163 samples from China, Myanmar, and personal import combined (Table 4). The average quantity of API in all the samples was 98.1% ± 2.7 (mean ± Standard Deviation-SD) of the label claim. Content uniformity for all samples was within the compliance range (AV value was below or equal to 15). Figure 1a, b, and c shows the frequency of the mean API in the quantity test of all samples collected between 2012 and 2015.

**Table 4:** Summary of the results of laboratory analysis

Sampling Site	Year	Test	Test (n/%)				
			Originator Brand		Generic		Total samples, n
			Compliant	Non-compliant	Compliant	Non-compliant	
China (Shanghai)	2012	Potency	9/100	0/0	34/97	1/3	44
		Content Uniformity	9/100	0/0	35/100	0/0	
		Dissolution	9/100	0/0	35/100	0/0	
Personal import samples	2013	Potency	19/100	0/0	13/100^a^	13/100^a^	59
		Content Uniformity	19/100	0/0	13/100^a^	13/100^a^	
		Dissolution	19/100	0/0	8/62^a^	5/38^a^	
Yangon, Myanmar	2015	Potency	1/100	0/0	59/98	1/2	60
		Content Uniformity	1/100	0/0	59/98	1/2	
		Dissolution	1/100	0/0	51/86	8/14	
**Total number of samples, n**			**163**				

^a^Among the *n* = 31 generic samples, only 13 samples could be fully tested, as the number of tablets was insufficient

**Fig. 2:**
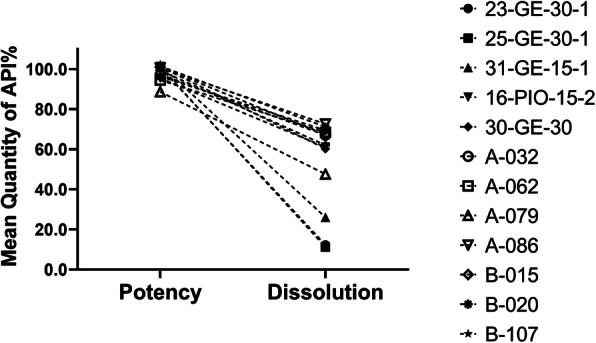
Mean Quantity of %API of the Non-Compliant Samples versus their Dissolution Rate in the Dissolution Medium

**Fig. 3:**
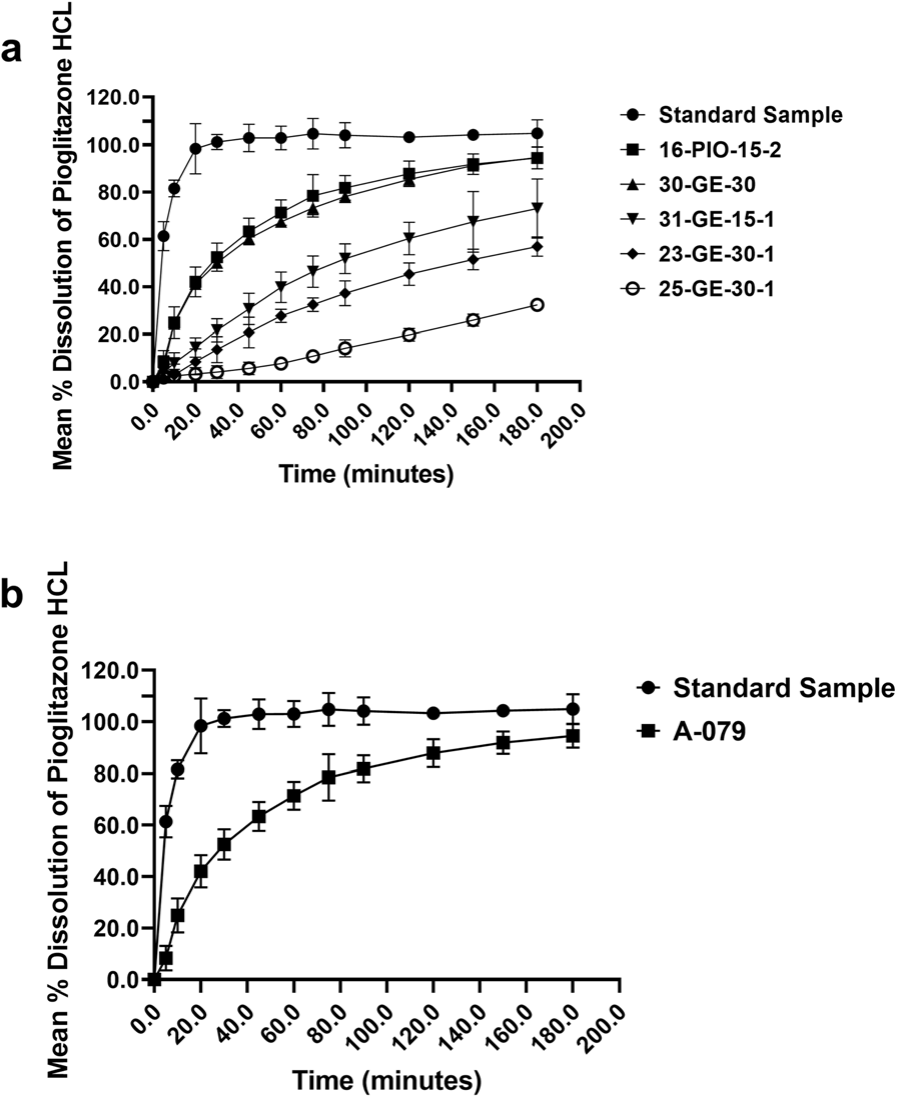
Dissolution Profile of Standard Sample and Non-Compliant Samples upto 180 min. **a** Personal Import Samples and **b** Myanmar Samples

However, In the case of the Myanmar and personal import samples, there was a major problem with dissolution. Figure 1d, e, and f shows the frequency of the mean API dissolved in the medium in the dissolution test. In the case of the personal import samples, *n* = 32 samples were analyzed for each manufacturer and batch number, and 15.6% failed to release the required amount of pioglitazone within the specified time. Among the *n* = 60 Myanmar samples, 13.3% were non-compliant in the dissolution test. The average percent release of the compliant samples was 95.0 ± 3.9 (mean ± SD). The average percent release of the non-compliant samples is shown in Table 5 and their quantity versus dissolution rate in the dissolution medium are presented in Fig. 2. Time course studies of drug release from the non-compliant samples confirmed that they did not meet the threshold requirement for dissolution time in the dissolution medium (Fig. 3a and b), and most did not disintegrate in the dissolution medium (Supplemental Figure 1 and Supplemental Figure 2).

**Table 5:** Average percent release of non-compliant samples in the dissolution test

Sample source	Sample code	Mean % release ± SD
Personal import	23-GE-30-1	12.3 ± 5.3
	25-GE-30-1	11.2 ± 0.5
	31-GE-15-1	26.1 ± 2.7
	16-PIO-15-2	61.09 ± 2.6
	30-GE-30	60.3 ± 2.2
Yangon, Myanmar	A-032	67.0 ± 7.6
	A-062	68.5 ± 6.9
	A-079	47.6 ± 5.8
	A-086	72.6 ± 5.0
	B-015	67.3 ± 10.0
	B-020	69.4 ± 6.3
	B-107	71.2 ± 11.5
	PA-013	62.0 ± 5.2

## Discussion

Our findings revealed serious issues with pioglitazone purchased via the internet and imported into Japan. According to the Pharmaceutical Affairs Law in Japan, selling prescription drugs without a prescription is prohibited, but among the *n* = 32 online pharmacy sites visited, none required a prescription to sell pioglitazone (Table 2). In addition, 45 mg pioglitazone is not approved for sale in Japan, but 4 pharmacies were selling this formulation. Also, 14 pharmacies were selling pioglitazone without any restriction on the amount purchased. We found that most of the non-compliant samples were obtained from sites that did not give any physical address (Table 2).

On the other hand, the Myanmar samples, which also showed a high failure rate (Tables 4 and 5, Fig. 3), were all of foreign origin, which may suggest that medicines can enter the country through unauthorized channels. The authenticity of the products and legitimacy of the manufacturers remained unclear due to the poor responses to our questionnaire (Supplemental Files S1 and S2) from both manufacturers and medicine regulatory authorities (Table 3), as observed previously [17, 38, 39]. A possible explanation of the low response in the former case might be that manufacturers are already aware that their products are of low quality. However, there is clearly a need to improve legitimacy verification as well [40, 41].

In the case of China, only 1 sample out of =52 failed to meet the pharmacopoeial requirement for API (Table 4). This may mean that quality of pioglitazone in China is better than has been suggested [22, 42, 43].

Poor dissolution was the predominant problem among non-compliant samples. Among personally imported samples, up to 8.5% were substandard (Table 4, Fig. 1e), and *n* = 3 released less than 30% of the required amount (Table 5, Fig. 2). Nevertheless, we could not establish whether these samples were falsified. Among the Myanmar samples, the prevalence was even higher, at 13.3% (Table 4, Fig. 2). The dissolution test is an important indicator in bioequivalence testing, e.g., to compare generic products with the parent drug. However, many studies have shown that there can be marked differences in dissolution times between originator brand and generic drugs [12, 44–46]. Time course studies of the non-complaint samples showed a marked differences in the dissolution behavior compared to the standard sample. Release rate of pioglitazone for most of these non-compliant samples were below the threshold limit even after 180 min in the dissolution medium (Fig. 3, Supplemental Figure 1 and Supplemental Figure 2). The ineffectiveness of these formulations could easily result in treatment failure. Drug dissolution testing is still being considered to be a minimal requisite in many pharmaceutical quality studies, given that the dissolution testing is one of the key parameter to observe the physicochemical changes in a solid formulation [47].

This study has several limitations. One minor limitation of this study is that the data are relatively old. Medicine regulatory authorities often show reluctance on data sharing and publication. There are also limitations and conditions on data sharing even if they approve and in many cases, it takes longer time than usual to get their final approval. However, the entire process is time consuming and eventually lead to the delayed publication, which is similar to this situation. Additionally, it deals only with a single drug, pioglitazone, collected by a cross-sectional method from specific areas of Myanmar and China, so our results may not reflect the situation in other regions of those countries. Therefore the results may not be directly comparable with other reported findings.

## Conclusion

Many studies still only focus on the content of API which can provide a sense of false security, as the samples are classified as ‘good quality’ without any information of dissolution/availability. High levels of substandard pioglitazone with poor dissolution properties were identified among samples purchased from online sites and personally imported into Japan, and also among samples collected in Myanmar, whereas only a single sample from China was non-compliant. The internet sites all failed to comply with Japanese law in various respects. Coordinated steps should be taken to ensure best practices including improvement of national and international regulatory oversight to address the situation.

## Supplementary Information

**Additional file 1.**

**Additional file 2.**

**Additional file 3.**

**Additional file 4: Supplemental Figure 1.** Photographs of the tablet of standard and undissolved non-compliant sample from Myanmar in the dissolution vessel. A) Standard sample (Actos) and B) non-compliant sample A-079.

**Additional file 5: Supplemental Figure 2.** Photographs of the tablet of standard and undissolved non-compliant samples from personal import in the dissolution vessel. A) Standard sample (Actos); B) 16-PIO-15-2; C) 23-GE-30-1; D) 25-GE-30-1; E) 26-GLI-15-1; F) 31-GE-15-1.

## Data Availability

All available data generated or analyzed during this study are included in this article and supplemental files. Raw analysis data for individual sample cannot be provided because of restrictions imposed the respective MRAs.

## Abbreviations

API: Active pharmaceutical ingredient
AV: Acceptance value
FDA: Food and Drug Administration
FIP: The International Pharmaceutical Federation
HPLC: High-performance liquid chromatography
INN: International non-proprietary name
JP: Japanese Pharmacopoeia
JPMA: Pharmaceutical Manufacturers Association of Japan
MHLW: Ministry of Health, Labour and Welfare
MRA: Medicine Regulatory Authority
NRA: National Regulatory Authority
RPM: Revolutions per minute
SD: Standard Deviation
SF: Substandard and Falsified
USA: United States of America
USP: United States Pharmacopoeia
UV: Ultra-violet
WHO: World Health Organization
