# Supplementary material for: Patient safety and public health concerns: poor dissolution rate of pioglitazone tablets obtained from China, Myanmar and internet sites
Source: BMC Pharmacol Toxicol. 2021 Mar 2;22:12. doi: 10.1186/s40360-021-00478-x (PMC7923830; doi:10.1186/s40360-021-00478-x)
Supplement: Supplementary file 1 — Additional file 1. [file 40360_2021_478_MOESM1_ESM.pdf]

SAMPLE CODE:

SAMPLE AUTHENTICATION

	Sample's description			Pl. check appropriate box, if the information provided is identical to your genuine sample	
1	Trade Name			☐Yes	☐No
2	Active Ingredient & Strength			☐Yes	☐No
3	Dosage Form			☐Yes	☐No
4	Manufacturer's Name			☐Yes	☐No
5	Manufacturer's Address			☐Yes	☐No
6	Batch/Lot Number:			☐Yes	☐No
7	Manufacturing Date:			☐Yes	☐No
8	Expiry Date:			☐Yes	☐No
9	Distributor's Name			☐Yes	☐No
10	Distributor's Country			☐Yes	☐No
11	Registration No.			☐Yes	☐No
12	Manufacturing License Number			☐Yes	☐No
13	Is the logo authentic?			☐Yes	☐No
14	Is the trade name written appropriately (font, spell, ®)?		font	☐Yes	☐No
			spell	☐Yes	☐No
			®	☐Yes	☐No
15	Are the active ingredient(s) name(s) written appropriately?			☐Yes	☐No
16	Does the physical characteristics of the dosage form are uniform and consistent?		form	☐Yes	☐No
			shape	☐Yes	☐No
			color	☐Yes	☐No
			coating	☐Yes	☐No
			size	☐Yes	☐No
17	Is the product under this dosage form registered and authorized for sale in Country Name?			☐Yes	☐No
18	Please write correct information in the space provided below, if you checked 'No' to any of the above points (1-17)				

19	Is this medicine Genuine or Falsified?	☐**Genuine** ☐**Falsified**
20	If you checked 'Falsified' for the above question, please indicate the details about the difference of Genuine product and the Falsified one.	

MARKETING IN SAMPLING COUNTRY			
21	Is the sample medicine approved by the Drug Regulatory Authority in ***manufacturing country***?		
	☐Yes / Provide approval / registration number；______________________<br>☐No		
22	Is the sample medicine approved for marketing in ***Country Name***?	☐Yes	☐No
23	If you checked 'No' to the above question, please answer following two additional questions:		
	i. Please write the countries where this medicine is approved for marketing.		
	ii. Do you know that this medicine is sold in ***Country Name***?	☐Yes	☐No

Pictures of Sample:
