# Supplementary material for: Patient safety and public health concerns: poor dissolution rate of pioglitazone tablets obtained from China, Myanmar and internet sites
Source: BMC Pharmacol Toxicol. 2021 Mar 2;22:12. doi: 10.1186/s40360-021-00478-x (PMC7923830; doi:10.1186/s40360-021-00478-x)
Supplement: Supplementary file 2 — Additional file 2. [file 40360_2021_478_MOESM2_ESM.pdf]

MEDICINE AUTHENTICATION FORM

For Drug Regulatory Authority of ***Manufacturing Country***

Please provide necessary information for each of the manufacturers and their medicine products mentioned below. If you have additional information that might be important to judge whether the medicine is falsified or not, please indicate such in the remarks column.

**Name of the Manufacturer:**<br>**Country:**	
1. Is this manufacturer licensed by the Drug Regulatory Authority of your country?	☐Yes ☐No
2. If Yes, please mention manufacturer's License Number:	
3. Is this a GMP qualified manufacturer of your country?	☐Yes ☐No
**Products**	
Please check an appropriate box, if the regulatory authority of your country approves the manufacturer to produce mentioned medicine(s).	

Trade Name, strength, form	Active ingredient	Approval status	Remarks (if any)
		☐Yes ☐No	
