# Supplementary material for: Patient safety and public health concerns: poor dissolution rate of pioglitazone tablets obtained from China, Myanmar and internet sites
Source: BMC Pharmacol Toxicol. 2021 Mar 2;22:12. doi: 10.1186/s40360-021-00478-x (PMC7923830; doi:10.1186/s40360-021-00478-x)
Supplement: Supplementary file 3 — Additional file 3. [file 40360_2021_478_MOESM3_ESM.pdf]

Registration Verification

For ***Name of MRA***, ***Ministry of Name of Ministry***, ***Country Name***, ***Year***

Name of the Medicine

Please check appropriate boxes ☑ on confirming registration of the manufacturers and their products listed below. If the registration number does not exist, please check □No in Registration column. If the registration number is old, please write down the new number against each product in Comments column. In case the registered package size is different from the registration or you have any comments, please write down in Comments column.

Manufacturer's Name	Registered	Code	Brand Name /Strength (package size)	Labeled registration Number	Registration	Comments
**Country Name**						
Manufacturer Name	□ Yes<br>□ No	Sample Code			□ Yes<br>□ No	□ This is old number<br>New number_____________<br>□ Registered package size is different<br>________________________<br>□ Any comments
