# Supplementary figures and images for: Patient safety and public health concerns: poor dissolution rate of pioglitazone tablets obtained from China, Myanmar and internet sites
Source: BMC Pharmacol Toxicol. 2021 Mar 2;22:12. doi: 10.1186/s40360-021-00478-x (PMC7923830; doi:10.1186/s40360-021-00478-x)

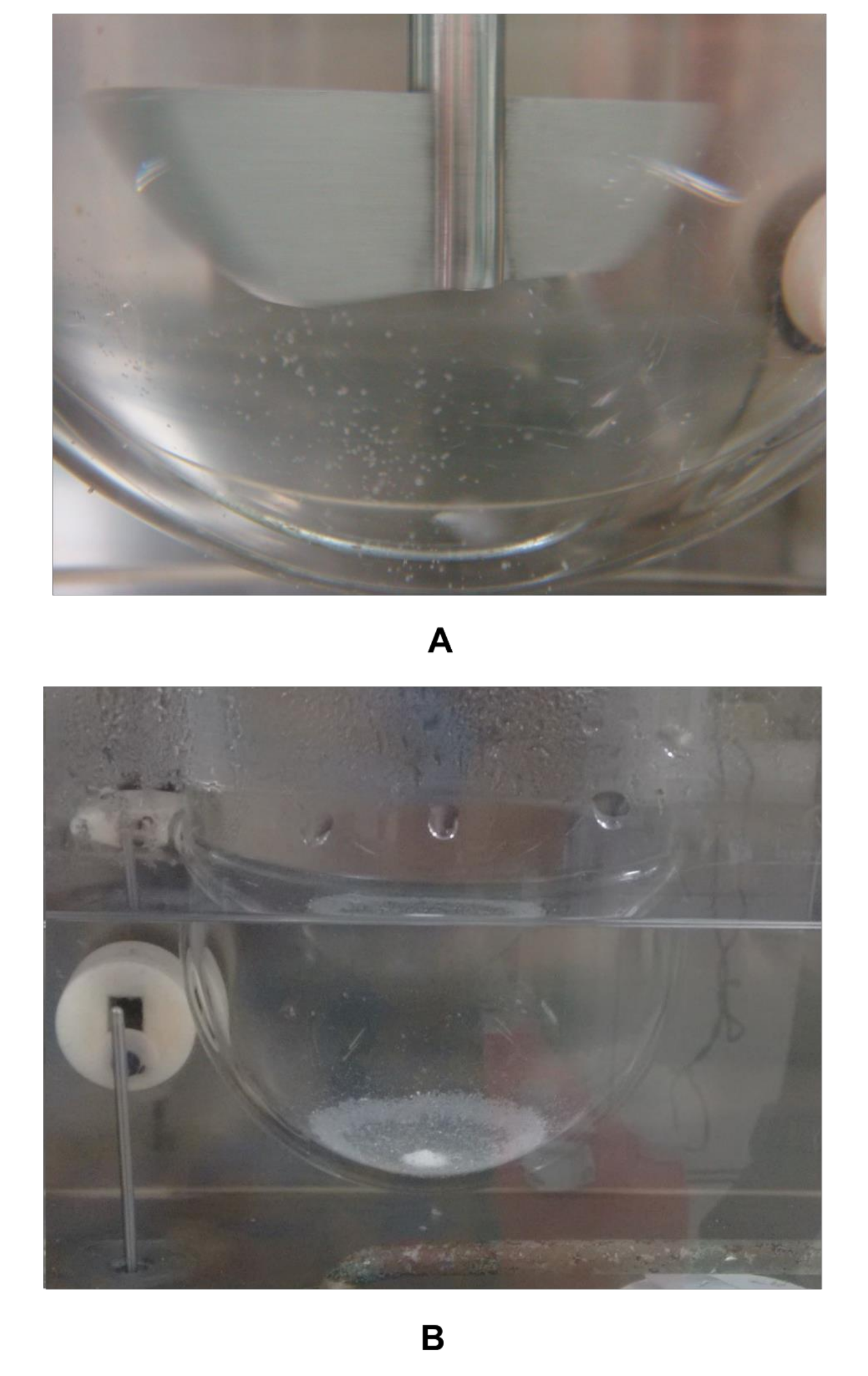

Supplement: Supplementary file 4 — Additional file 4: Supplemental Figure 1. Photographs of the tablet of standard and undissolved non-compliant sample from Myanmar in the dissolution vessel. A) Standard sample (Actos) and B) non-compliant sample A-079. [file 40360_2021_478_MOESM4_ESM.tif]

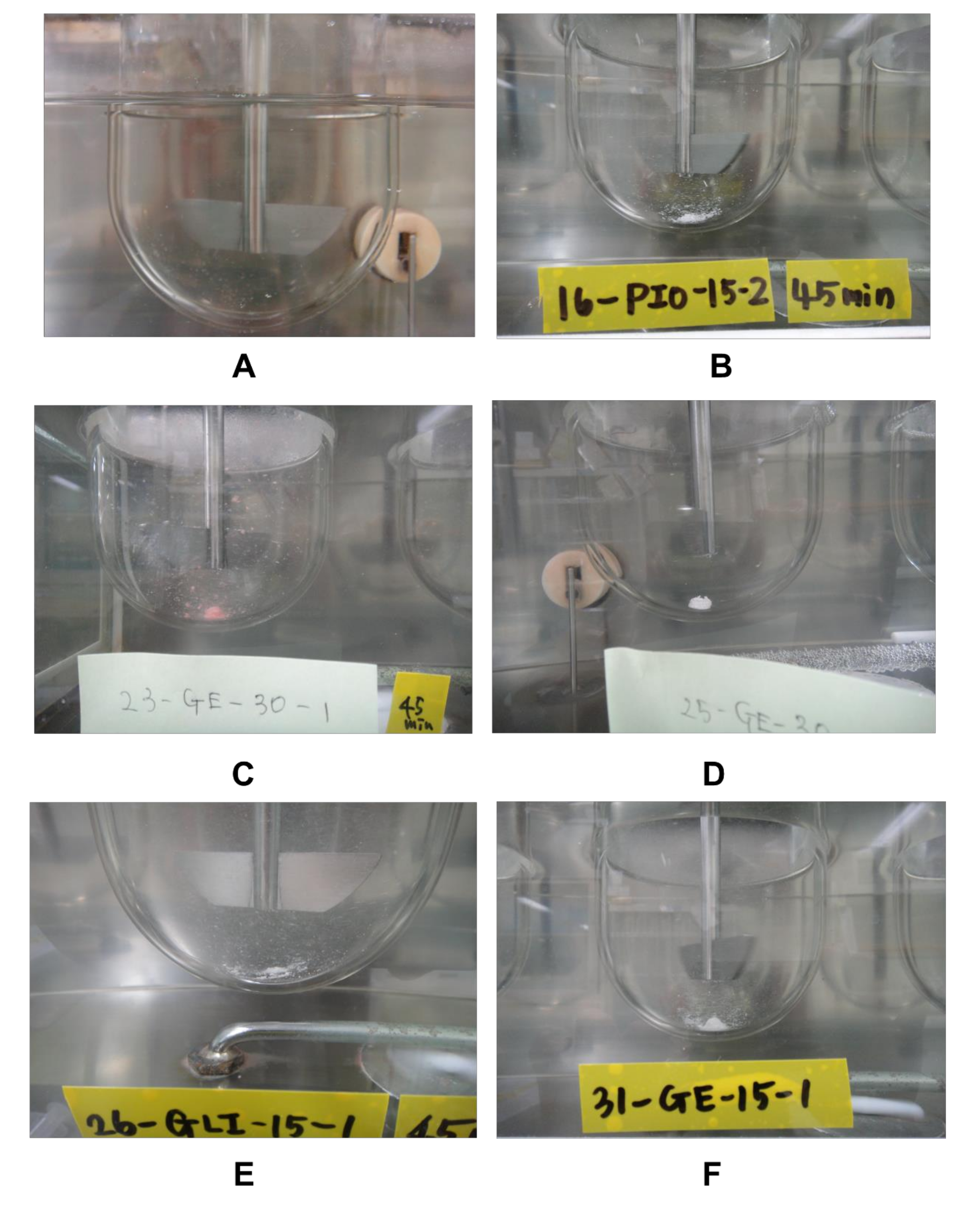

Supplement: Supplementary file 5 — Additional file 5: Supplemental Figure 2. Photographs of the tablet of standard and undissolved non-compliant samples from personal import in the dissolution vessel. A) Standard sample (Actos); B) 16-PIO-15-2; C) 23-GE-30-1; D) 25-GE-30-1; E) 26-GLI-15-1; F) 31-GE-15-1. [file 40360_2021_478_MOESM5_ESM.tif]
